# Effects of comprehensive cardiac rehabilitation on functional capacity and cardiovascular risk factors in Brazilians assisted by public health care: protocol for a randomized controlled trial

**DOI:** 10.1590/bjpt-rbf.2014.0192

**Published:** 2016-11-16

**Authors:** Gabriela S. S. Chaves, Gabriela L. M. Ghisi, Sherry L. Grace, Paul Oh, Antonio L. Ribeiro, Raquel R. Britto

**Affiliations:** 1 Departamento de Fisioterapia, Universidade Federal de Minas Gerais (UFMG), Belo Horizonte, MG, Brazil; 2 Cardiovascular Prevention and Rehabilitation Program, University Health Network (UHN), Toronto, ON, Canada; 3 School of Kinesiology and Health Science, York University, Toronto, ON, Canada; 4 Divisão de Cardiologia e Cirurgia Cardiovascular, Hospital Universitário, Universidade Federal de Minas Gerais (UFMG), Belo Horizonte, MG, Brazil

**Keywords:** coronary artery disease, rehabilitation, patient education, randomized controlled trials, protocol

## Abstract

**Background:**

Cardiovascular Disease (CVD) is the leading burden of disease worldwide. Moreover, CVD-related death rates are considered an epidemic in low- and middle-income countries (LMICs). Research shows that cardiac rehabilitation (CR) participation reduces death and improves disability and quality of life. Given the growing epidemic of CVD in LMICs and the insufficient evidence about CR programs in these countries, a Randomized Control Trial (RCT) in Latin America is warranted.

**Objective:**

To investigate the effects of comprehensive CR on functional capacity and cardiovascular risk factors.

**Method:**

The design is a single-blinded RCT with three parallel arms: comprehensive CR (exercise + education) versus exercise-based CR versus wait-list control (no CR). The primary outcome will be measured by the Incremental Shuttle Walk Test. Secondary outcomes are risk factors (blood pressure, dyslipidemia, dysglycemia, body mass index and waist circumference); tertiary outcomes are heart health behaviors (exercise, medication adherence, diet, and smoking), knowledge, and depressive symptoms. The CR program is six months in duration. Participants randomized to exercise-based CR will receive 24 weeks of exercise classes. The comprehensive CR group will also receive 24 educational sessions, including a workbook. Every outcome will be assessed at baseline and 6-months later, and mortality will be ascertained at six months and one year.

**Conclusion:**

This will be the first RCT to establish the effects of CR in Latin America. If positive, results will be used to promote broader implementation of comprehensive CR and patient access in the region and to inform a larger-scale trial powered for mortality.

## BULLET POINTS

- CVD is growing in low- and middle-income countries (LMICs).
- Cardiac Rehabilitation (CR) programs are grossly underdeveloped.
- There is insufficient evidence about CR programs in LMICs.
- The effects of comprehensive CR will be evaluated in an RCT.
- Results will be used to improve care of cardiac patients in Brazil.

## Introduction

The most prevalent non-communicable disease globally, namely cardiovascular disease (CVD), is also the leading cause of mortality, with over 80% of the deaths occurring in low- and middle-income countries (LMICs)^1^. For example, in the MIC of Brazil, CVD is also the leading cause of death. Specifically, CVD was responsible for 29% of total mortality in 2010 (approximately 37,000 deaths/year)^2^. CVD requires appropriate long-term management if further acute events and other complications are to be reduced or avoided.

Cardiac Rehabilitation (CR) is a chronic disease management program delivering structured exercise, patient education to promote behavioral changes and psychological support to decrease risk factor burden and, thus, optimize secondary prevention^3^. CR is an essential part of outpatient CVD care and is a Class I recommendation in CVD clinical practice guidelines^4^. Participation in CR has been shown to reduce morbidity and mortality by approximately 25% when compared to usual care in high-income countries^3^. Robust evidence demonstrates participation also improves functional capacity, as well as risk factor burden, knowledge, heart health behaviors, and psychosocial well-being^3^. It is also demonstrated to be cost-effective and “value for money”^5^.

Despite its benefits and the burgeoning implementation of expensive interventional cardiology in LMICs (which are only accessible to approximately 40% of Brazilians in the private health system), CR is grossly underdeveloped^6^. Programs are limited to major metropolitan centers and focus primarily on exercise training to the exclusion of other recognized “core components”^7^ of CR such as patient education and counseling^6^ ^,^ ^8^. Indeed, systematic reviews^9^ ^,^ ^10^ have demonstrated the positive effects of patient education on behavior change in CVD patients. Research by our group has demonstrated the benefits of education in CR through increasing patients’ knowledge and facilitating behavior change^11^.

It is estimated that only 14% of patients access CR in MICs such as Brazil, where it is estimated there are only 39 programs nationally^12^. While the availability of CR programs in the majority of Latin American countries is just beginning to be known, their broader implementation is hampered by lack of evidence demonstrating benefits in these lower-resource settings^12^. A recent review of randomized controlled trials (RCTs) of CR in LMICs identified only two RCTs, neither of which was undertaken in Latin America^13^. To support broader CR delivery and service reimbursement, we must robustly demonstrate that CR is beneficial in this context. Accordingly, the objectives of this trial are to investigate pragmatically whether participation in a comprehensive CR program (i.e., exercise with education) in a Latin American MIC results in better functional capacity, cardiovascular risk factor control, health behavior, disease-related knowledge, depressive symptoms, and lower mortality when compared to exercise-only CR or wait-list control. It is hypothesized that participants randomized to comprehensive CR will have significantly better outcomes than those participating in exercise-based CR or not participating.

## Method

### Study design

The design is a single-blinded, single-site, pragmatic, superiority RCT with three parallel arms: comprehensive CR (education and exercise) *versus* exercise-based CR (no education, as delivered in Brazil) *versus* wait-list control (i.e., no CR). Patient assessments will be undertaken pre-randomization and again six months later (in accordance with the end of CR). Mortality will be ascertained at six months and one year post-recruitment. The study was submitted to Plataforma Brasil, and the research ethics approval has been secured from Universidade Federal de Minas Gerais (UFMG), Belo Horizonte, MG, Brazil (898.235) and York University, Toronto, ON, Canada (e2015-172).

### Setting

This RCT will be conducted in the Brazilian city of Belo Horizonte (capital of the state of Minas Gerais). Brazil was chosen as the initial MIC setting for testing because of (1) the great burden of CVD^2^, (2) the availability of country-specific CR guidelines (the only CR guidelines developed in any MICs are in South America and China)^6^, and (3) there has never been an RCT of CR (with any outcome) in South America to our knowledge^10^.

The program where the trial will be conducted is called **“**The Acute Myocardial Infarction (AMI) Management System”, which was established in 2009. It was developed to reduce the high level of mortality from AMI in the region, around 16.2%^2^. The program provides a continuum of care for AMI patients from emergency care through to secondary prevention six months after the event. The program includes CR for interested patients; however, this has never been evaluated.

### Participants

Patients will be approached at the established AMI public healthcare system of the City of Belo Horizonte, Brazil. Coronary artery disease or post-MI patients or those who have undergone percutaneous coronary intervention or coronary artery bypass grafting and have been referred to CR will be eligible to participate in the study. The inclusion criteria are patients older than 18 years old and living in the Belo Horizonte area. The exclusion criteria are any comorbid physical or serious mental condition which would interfere with the ability to exercise according to CR clinical practice guidelines^14^ (i.e., heart failure with ejection fraction less than 45%, complex ventricular dysrhythmia, advanced dementia, leg amputation, advanced cancer, disabling stroke, Parkinson’s disease, or substance dependence) or any visual or cognitive condition which would preclude the participant from completing the questionnaires.

### Intervention arms

The CR program was developed based on Canadian^4^ and Brazilian^15^ CR guidelines. It is delivered by a multidisciplinary team including physical therapists, dietitians, nurses, and physical educators. The program is exercise-based, not comprehensive. It is offered at no charge to patients. Participants undergo an assessment including risk-factor assessment at intake and again at end of the program.

The main program is six months in duration, with 36 1-hour exercise sessions offered at the following frequencies:

1. participants come to CR for 12 sessions, three times a week (total of four weeks of intervention).
2. participants come to CR for eight sessions, twice a week (total of four weeks of intervention).
3. participants come to CR for 16 sessions, once a week (total of 16 weeks of intervention).

Each participant will receive an individualized exercise prescription based on a grade exercise stress test. Participants will be exercising between 50 and 80% of heart rate reserve. At all stages of the program, patients will be requested to exercise in their community other days of the week, to accumulate 30 or more minutes of physical activity at a moderate to vigorous-intensity five or more days per week, as recommended in the guidelines^4^ ^,^ ^14^ ^,^ ^15^.

In the comprehensive CR arm, 24 education sessions will be offered, each with 30 minutes duration. More specifically, the education component consists of:

- weekly group education sessions, which are strategically mapped based on patients’ information needs and sequenced to support the program learning outcomes^16^. Education sessions are delivered by a health educator team. See Table 1 for the content of the educational curriculum.
- a comprehensive education workbook to accompany the sessions, containing 20 chapters. The empirically validated English version^16^ ^,^ ^17^ has been translated and culturally adapted to Brazilian Portuguese. Clinicians and patients have reviewed the material, and a plain language and clear design review was completed in preparation for this trial.

**Table 1:** Sections of the educational curriculum for CR patients.

**Period (weeks)**	**Section**	**Description**
**Beginning** **(weeks 1-5)**	Safety and Foundation	Information about the CR program, their aerobic exercise prescription and safety, managing angina, irregular heartbeats, diabetes, exercising in cold and hot weather, the heart (anatomy, pathophysiology, diagnoses, and treatment) and cardiac medications.
**Middle** **(weeks 6-12)**	Skill Development	Information about the patient’s risk factor profile, goal setting and action planning, resistance training, nutrition (fats, fiber, reading food labels, sodium), psychosocial risk, and sexual intimacy.
**End** **(weeks 18-24)**	Preparing for Graduation	Information about how much physical activity is good, aerobic and resistance training progression, relapse planning, and graduation.

CR: cardiac rehabilitation.

The standard of care for Brazilian adults with CVD does not include access to CR for all patients, given the gross lack of capacity^6^. All participants will have follow-up appointments with their physician as deemed medically appropriate. Consistent with CONSORT guidelines, usual care will be described in detail for each participant (e.g., number of health visits - both inpatient and outpatient, other treatments). Participants randomized to the wait-list control arm will receive CR after 6-month mortality is ascertained.

### Procedure

**Figure 1:**
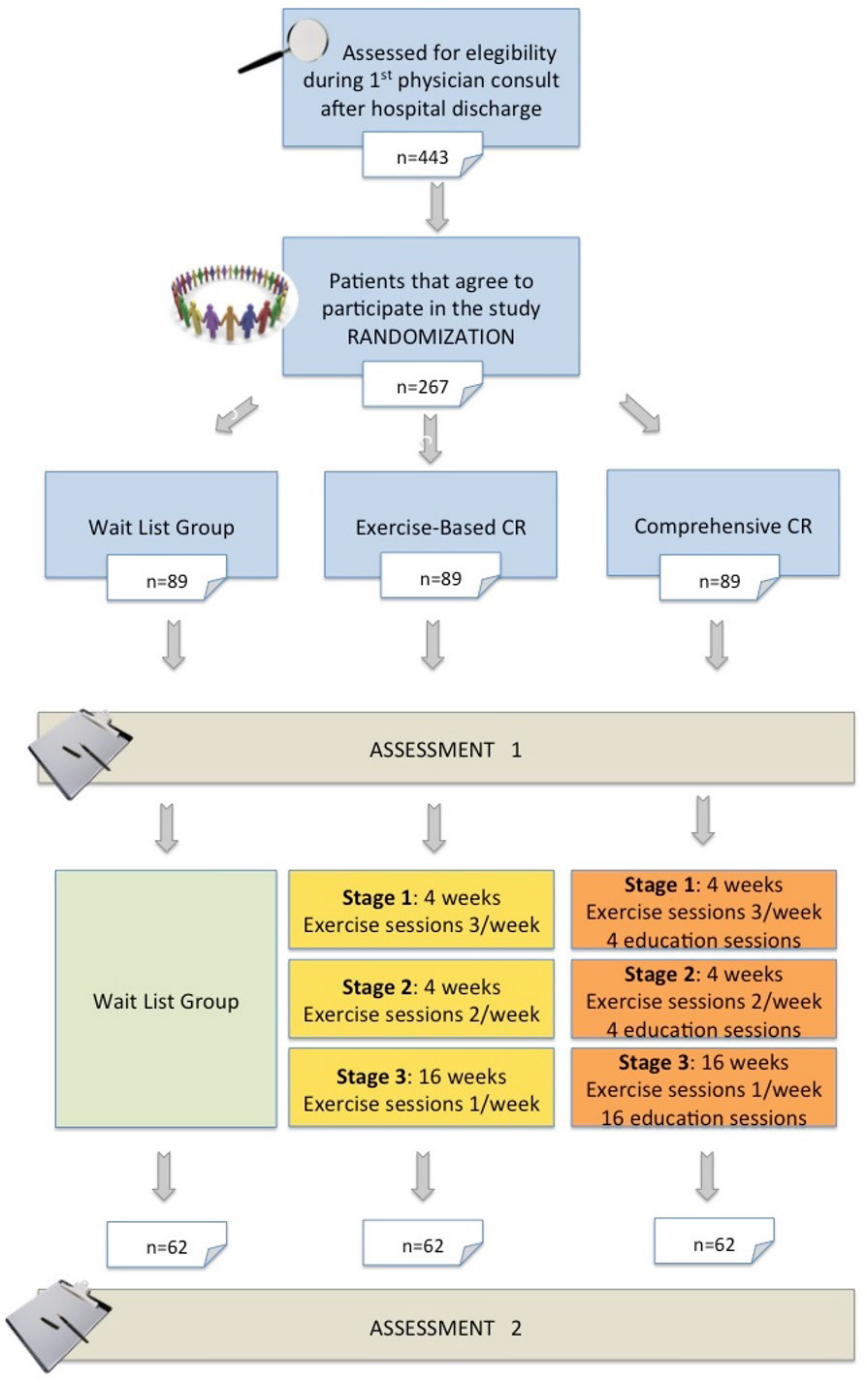
Flow diagram of the trial.

See Figure 1 for the trial’s flow diagram. Consecutive patients will be approached by a doctoral student during the first physician consult after hospital discharge if the patient is interested in learning about the study. The number of patients approached and date will be recorded, as well as the reasons for inclusion/exclusion. With informed written consent from the patient and CR clearance from the physician, potentially eligible participants will be scheduled to come on-site to complete pre-test assessments. Participants will be asked to complete a sociodemographic questionnaire to establish the generalizability of the sample, among other surveys. Clinical information will be extracted from participants’ charts.

Eligible participants will be randomized to one of the three groups. The randomization sequence was generated using the randomization.com website in random blocks of four, with a 1:1:1 allocation ratio. To ensure allocation concealment, the local principal investigator (RB) has the allocation sequence in a password-protected file and will only provide randomization information to the student once it is confirmed the participant is eligible. Due to the nature of the intervention, participants and the doctoral student cannot be blind to treatment allocation.

The primary outcome of functional capacity and all other outcomes will be assessed again at six months post-randomization. Mortality will be ascertained from hospital charts and family phone call at six months and one year. With regard to the 6-month assessment, participants will be invited to come to the study center to: (1) undertake the shuttle walk test as the indicator of functional capacity, (2) undertake assessments of secondary outcomes including a blood draw for lipids, and (3) complete surveys related to tertiary outcomes.

A master’s student blinded to random allocation will undertake post-test assessments, outcome ascertainment, and data entry. To minimize loss to follow-up, we will telephone reminders for patients to come on-site for these assessments.

### Measures

Participants will be asked to complete a sociodemographic questionnaire. Clinical characteristics will be extracted from the medical charts, including sex, age, risk factors, cardiac history, cardiac test results, comorbidities, and medications. CR session attendance will be extracted from program charts at post-test for participants randomized to the CR arms.

### Primary outcome: functional capacity

The primary outcome of this trial was carefully chosen. Functional capacity is not only important to an individual in terms of independent living and quality of life; it is also strongly related to health outcomes, including a graded inverse relationship with mortality^18^.

At pre-test and 6-months later, the Incremental Shuttle walk test (ISWT)^19^ will be performed. The ISWT consists of an incremental, walking test where participants are required to walk up and down a 10-meter course. The speed of walking, which is increased by a small increment every minute (0.17 ms-l), is externally paced and controlled by audio signals played from a tape recorder^19^. There are 12 levels in total, beginning with 0.5 ms^–1^, and each level lasts for one minute. At the end of each minute, exercise heart rate (HR) and rating of perceived exertion (RPE) scores will be recorded. The test is terminated if participants feel too breathless or fatigued to maintain the required speed to complete a 10-meter shuttle interval in the time allowed. After the test, the number of completed shuttles will be recorded and the total distance ambulated will be computed. Peak RPE, peak HR, time to exhaustion, and the reason(s) for test termination will also be recorded.

### Secondary outcomes: risk factors

Blood pressure will be assessed using the validated 7670-06 mobile stand (Welch Allyn Inc., Skaneateles Falls, NY, USA). Mean systolic and diastolic blood pressure values will be recorded and hypertension will be considered where values exceed 140/90 mmHg^14^. A weight scale and measuring tape are available at the center and will be used to assess anthropometrics. Those with body mass index above 30 kg/m^2^ will be considered obese^14^. Waist circumference will be assessed at the superior border of the iliac crest, in accordance with standardized guideline^14^. Values greater than 102 cm in men and 88 cm in women will be considered indicative of central obesity^20^. Glycaemia and lipid values will be extracted from center charts. Diabetes will be considered present where fasting blood glucose exceeds 126 mg/dl and dyslipidemia will be considered present where total cholesterol values exceed 240 mg/dl^14^.

### Tertiary outcomes

#### Heart health behaviors

Smoking will be assessed via self-report (current, never, former). Other behaviors will be assessed using psychometrically validated scales, as outlined below.

Exercise: Participants will be asked to wear a pedometer for seven days at pre- and post-test and to record their daily steps on a log. Exercise will also be assessed using the Brazilian Portuguese version of The Godin-Shephard Leisure-Time Physical Activity Questionnaire^21^, which is a self-administered questionnaire that assesses the frequency and intensity of physical activity performed in a week. The respondents report the number of times they engaged in vigorous, moderate and light intensity physical activity for at least 15 minutes, considering a usual period of seven days. The frequency indicated by the individual is multiplied by a specific coefficient, which corresponds to the energy expenditure in metabolic equivalents of task (MET). Higher scores indicate higher levels of physical activity during leisure^21^.
Medication adherence: Cardiac medication adherence will be assessed using the Brazilian Portuguese version of the Morisky Medication Adherence Scale (MMAS-8)^22^. The scale contains seven questions with closed dichotomous (yes/no) response options and the last question is scored on a 5-point Likert from “never” to “always”. Scores are totaled, with higher scores indicating greater adherence, and scores above the cut-off of 6 considered “adherence”.
Diet: Diet will be assessed using the 14-item Food Frequency Questionnaire (FFQ) for Cardiovascular Prevention^23^, which was designed to assess the consumption of foods associated with an increase or decrease in coronary risk. A score is attributed to each food group, weighed according to their influence on coronary risk ranging from –36 to +47^23^.
Depressive symptoms: Depressive symptoms will be measured using the Patient Health Questionnaire-9 (PHQ-9), which is a brief screening instrument. Frequencies of symptoms of major depression are solicited from patients, yielding scores ranging from 0 to 27, with higher scores indicating more severe symptoms. Severity categorizations are specified, with scores above 10 generally accepted as “elevated”. The PHQ-9 has been shown to have reasonable sensitivity and specificity for patients with CVD^24^. This instrument has been translated and validated to Portuguese^25^.
Knowledge: Patients’ knowledge about their condition will be assessed using the Coronary Artery Disease Education Questionnaire II (CADE-Q II)^26^. The CADE-Q II is a 31-item test that assesses the cardiac patients’ level of knowledge about their medical condition, risk factors, exercise, nutrition, and psychosocial risk. Each item has four response options, and responses are scored from 0 to 3 (i.e., 3- fully correct answer; 1- partially correct answer; 0- wrong answer; and ‘I do not know’ response). The maximum total CADE-Q II score is 93. For the purposes of this trial, the CADE-Q II is being psychometrically validated in Brazilian Portuguese prior to administration.
Mortality: According to the 2011 Cochrane review by Heran et al.^27^, a trend towards a reduction in total mortality is observed in CR trials with 6-12 months follow-up. For this reason, we will ascertain mortality at six months and one year. Given the lower rates of screening and treatment of CV risk factors in the middle-income setting, we expect mortality rates and treatment effects to be higher than in high-income countries from where the RCTs summarized in the Cochrane review stemmed.

This data will be obtained from the center’s health system records and will be supplemented by family report via phone to capture deaths that may have occurred outside the hospital. Reason for death will be recorded where available.

### Sample size calculation

Based on previous studies, we considered a clinically important difference of 70 meters^28^ and assumed a standard deviation of 139 meters^29^. Using these values, we would require 62 participants per group to ensure 80% power at the 5% significance level to detect a statistically significant difference in our primary outcome of functional capacity (i.e., distance walked in the Shuttle Test)^30^. Conservatively assuming a 60% recruitment rate and a 70% retention rate (taking into consideration attrition due to death and loss to follow-up), we would need to approach 443 patients to achieve a sample of 267 at pretest and 186 at post-test (i.e., 62 per group).

### Statistical analyses

First, session attendance of participants in the two CR arms will be explored as a manipulation check. Second, differences in participants’ sociodemographic and clinical characteristics will be compared between groups to identify any chance difference that may have occurred despite random assignment, using chi-square and analysis of variance as appropriate. Third, retention rate will be computed, and differences in the sociodemographic and clinical characteristics of participants retained versus lost to follow-up will be compared using chi-square and t-tests as appropriate.

All statistical analyses will be performed considering the intention-to-treat analysis and a per protocol basis to mitigate bias. For the primary outcome of functional capacity, an analysis of covariance (ANCOVA) will be performed, with group (i.e., comprehensive CR *versus* exercise-based CR *versus* wait-list control) and pre-test distance as the independent variables and the distance on the shuttle walk test at post-test as the dependent variable, adjusting for any clinical and sociodemographic biases based on retention. For risk factors, ANCOVAs will also be computed with post-test scores as the dependent variable, experimental group as the independent variable, and pre-test scores as covariates. Post-hoc tests will be performed where significant group differences are observed. A Bonferroni correction will be applied as there are multiple secondary outcomes.

A similar approach will be used to assess group differences in health behaviors, depressive symptoms and knowledge (except for smoking where a logistic regression model will be run). Finally, a chi-square test will be performed to evaluate mortality by group.

## Discussion

It is anticipated that this trial will confirm the beneficial effects of CR in Brazil; however, the trial has not been powered for the tertiary outcome of mortality. To convince payers, namely governments and private insurance companies, to reimburse CR services, robust evidence demonstrating the CR participation results in reduced mortality, as well as unplanned readmissions would be compelling. Therefore, the results of the trial, if positive, will be used as the basis for sample size calculations for a larger trial powered to test the effects of CR (comprehensive or otherwise) on mortality, with re-hospitalizations as a secondary outcome. This trial would likely need to be multi-center to achieve the required sample size.

This trial as designed does have some limitations. First, it is a single-site study, which may limit generalizability. Potential addition of a second site is currently under consideration. Moreover, it is being undertaken in a publically funded center. The majority of Brazilians receive care in publically funded institutions increasing generalizability, but caution will be warranted in generalizing results to patients receiving cardiac care in privately funded settings. The benefit of undertaking the trial in the publically funded system is that all participants can receive the CR intervention without financial barriers. On a related note, as we are evaluating patients assisted by the public healthcare system, many will have low educational attainment and may have difficulty comprehending the questionnaires. Study staff will administer the scales to participants verbally in instances of low literacy or numeracy.

Third, participants may not fully adhere to the CR sessions; however, session attendance will be extracted from CR charts so that this can be considered in statistical analysis if appropriate. Fourth, there is no administrative source of data to ascertain our tertiary outcome of mortality, i.e., when patients die outside the clinical center where the trial is being undertaken. Unfortunately, many LMICs do not have strong systems of vital registration. For this reason as well, cause of death may not be ascertainable, and hence only total or all-cause mortality will be reported for the purposes of this trial. Nevertheless, mortality ascertainment via the center and family report should capture all events. Finally, the primary and secondary trial outcomes are all surrogate. If we observe improvements in functional capacity and risk factors, this will suggest potential mechanisms by which CR might improve survival and morbidity. There have been no CR trials in LMICs with mortality as an outcome and therefore this trial is a necessary preliminary step to achieving that.

Overall, if positive, the results of this trial will be used to advocate for greater implementation of CR and hence improve the care of cardiac patients in Brazil more broadly, as well as in other Latin American countries.

## Trial status

Recruitment started March 2015. Between March 2015 and September 2015, 41 patients have consented to participate in this trial. Post-test assessments are now commencing. Recruitment has been slower than anticipated due to a strike by cardiopulmonary technicians.
